# Whole brain dynamics during optogenetic self-stimulation of the medial prefrontal cortex in mice

**DOI:** 10.1038/s42003-020-01612-x

**Published:** 2021-01-14

**Authors:** Christopher G. Cover, Andrew J. Kesner, Shehzad Ukani, Elliot A. Stein, Satoshi Ikemoto, Yihong Yang, Hanbing Lu

**Affiliations:** 1 Neuroimaging Research Branch, National Institute on Drug Abuse, Intramural Research Program, NIH, Baltimore, MD 21224 USA; 2 Behavioral Neuroscience Research Branch, National Institute on Drug Abuse, Intramural Research Program, NIH, Baltimore, MD 21224 USA

**Keywords:** Functional magnetic resonance imaging, Motivation, Reward

## Abstract

Intracranial self-stimulation, in which an animal performs an operant response to receive regional brain electrical stimulation, is a widely used procedure to study motivated behavior. While local neuronal activity has long been measured immediately before or after the operant, imaging the whole brain in real-time remains a challenge. Herein we report a method that permits functional MRI (fMRI) of brain dynamics while mice are cued to perform an operant task: licking a spout to receive optogenetic stimulation to the medial prefrontal cortex (MPFC) during a cue ON, but not cue OFF. Licking during cue ON results in activation of a widely distributed network consistent with underlying MPFC projections, while licking during cue OFF (without optogenetic stimulation) leads to negative fMRI signal in brain regions involved in acute extinction. Noninvasive whole brain readout combined with circuit-specific neuromodulation opens an avenue for investigating adaptive behavior in both healthy and disease models.

## Abstract (web summary)

Cover et al. develop a method that permits functional MRI of brain dynamics while mice are cued to perform an operant task: licking a spout to receive optogenetic stimulation to the medial prefrontal cortex. This method presents noninvasive whole brain readout for adaptive behavior of mice in response to circuit-specific neuromodulation.

## Introduction

Functional magnetic resonance imaging (fMRI) can measure dynamic neural processes on a whole brain, systems level with excellent temporal and spatial resolution. Although it is relatively straightforward to acquire fMRI data while a human subject engages in a goal-directed task, it has proven extremely challenging to perform similar studies in rodents due to the difficulty in both limiting motion and mitigating stress during task performance. As a result, rodent fMRI studies are traditionally performed under anesthesia in the absence of any overt behavioral engagement. Additionally, though effective in motion reduction, anesthesia influences arousal, brain blood flow, metabolism and potentially affect neurovascular coupling mechanisms^1,2^.

Adaptive, goal-directed behavior (e.g., foraging for food or avoiding danger), a fundamental capacity for survival, requires serial complex actions guided by predictions informed by previous learning, which in turn depends on the dynamic integration of computational processing across multiple brain regions and systems^3^. The medial prefrontal cortex (MPFC), or Brodmann Area 25 in the human brain, is a major hub for such processes. While the homology of specific MPFC subregions between rodents and humans remains unsettled, rodent infralimbic cortex (ILc) is thought to be the homolog of the human subgenual anterior cingulate cortex (sgACC)^4^. It receives projections from the dorsomedial thalamic nucleus and hippocampus, while projecting to the nucleus accumbens, among other regions. It is a major constituent of the mesocorticolimbic dopamine pathway involved in reward, learning and decision making^5^. Dysregulation in this region has been implicated in various neuropsychiatric and substance abuse disorders^6,7^. Notably, sgACC is a prominent target in deep brain stimulation treatment for major depression^8^.

Stimulation of MPFC is known to be rewarding: that is, animals rapidly learn to voluntarily perform an operant behavior (e.g. a lever press) to receive MPFC stimulation^9^, a goal-directed behavior referred to as intracranial self-stimulation (ICSS) that was initially identified using electrical stimulation, which will activate all cellular elements (neurons, glia, fibers of passage) within the field of the electrode^10,11^. In contrast, emerging optogenetic techniques have enabled cell-type specific selective activation or inhibition. Optogenetic ICSS offers the opportunity to investigate how specific neural population connections form networks that drive a behavior^12^. We developed behavioral training paradigm in the context of fMRI paired with sucrose water to mitigate stress. We herein report the imaging platform and behavioral training procedures that allow for real time imaging of whole brain dynamic activity while a rodent operantly delivers circuit-specific optogenetic ICSS to the MPFC.

## Results

### Behavioral training for optogenetic ICSS

**Fig. 1:**
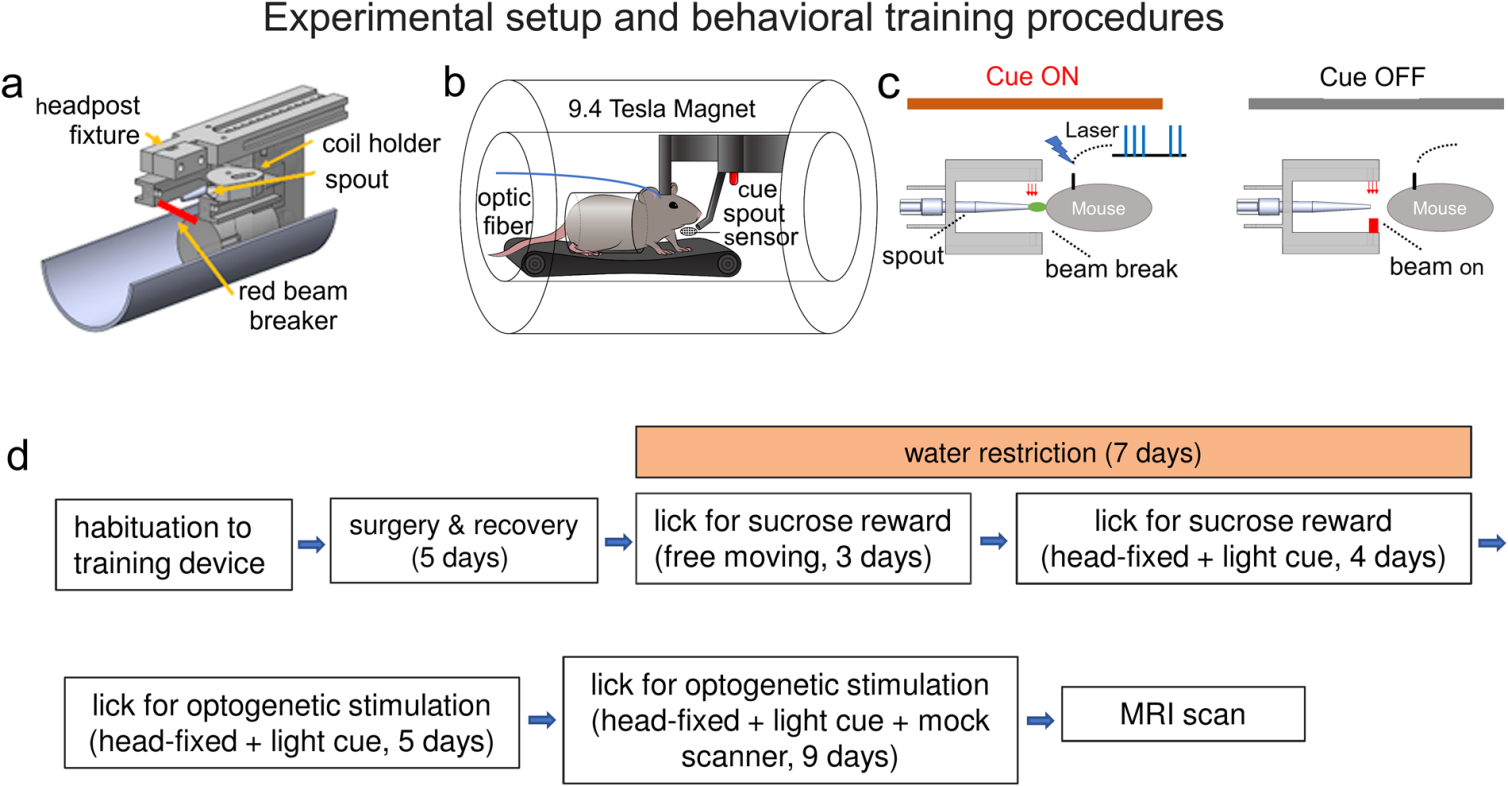
Illustration of the fMRI experimental setup and behavioral training procedures. **a**–**c** Illustration of the experimental setup to record fMRI during optogenetic MPFC self-stimulation in an awake mouse. A Laser pulse train is delivered when (i) the light cue is ON, and (ii) the mouse performs a spout lick, breaking a light beam that senses tongue movement. No Laser pulse is delivered when the cue light is OFF. **d** Experimental timeline illustrating the behavioral conditioning procedures.

FMRI acquisition generates strong acoustic noise and is very sensitive to motion artifacts. Minimizing head motion and reducing stress in this noisy environment are two major technical challenges to image awake, behaving rodents^13^. Our strategy was to train head-fixed mice to perform an operant reward task while also providing a supportive mechanism to help alleviate restraint stress inside the MRI scanner. Figure 1 illustrates the behavioral training paradigm and the conditioning procedures. Briefly, during a single acute surgical session we micro-injected channelorhodopsin-2 viral vector to the MPFC and implanted an MRI-compatible headpost onto the mouse skull. After recovery from surgery, mice (*n* = 10) were placed on a water-restricted schedule for one week^14^ and were subsequently trained to lick a spout to receive water supply. Animals were allowed for two hours of free water access after each training session to avoid dehydration. An optic lickometer was custom-designed to detect spout-licking behavior. Each spout-licking resulted in a photo beam break, triggering the delivery of 0.025 ml sucrose (10% by volume) via a syringe pump. The beam break and reward delivery were registered into a computer. The training progressed from an initial free-moving condition to the head-fixed condition.

A contextual cue light was subsequently introduced to indicate the availability of sucrose reward. The cue light turned on/off every 60 sec. Spout-licking only during the cue ON period resulted in sucrose delivery; licking during the cue OFF period had no programmed consequence. After animals successfully learned the association between the contextual cue and reward availability, as indicated by licking the spout more frequently during the ON period, the water restriction schedule was terminated and sucrose reward was replaced with optogenetic stimulation to the MPFC (herein referred to as opto-ICSS; parameters: 10 mW, 25 Hz, 4 pulses per pulse train, 5 ms pulse duration). Since the fMRI BOLD response is a relative quantity, and it requires a stable baseline condition as a reference against which to calculate activation. The introduction of the cue light established a period with fewer licking/opto-ICSS epochs, thus facilitating BOLD data processing. The duration of head-fixation progressively increased from an initial 15 min up to a total length of 60 min. Following consistent behavioral responding, mice were next habituated to the MRI imaging environment inside a mock scanner (which included representative MRI acoustic noise) while continuing to lick for opto-ICSS. We also introduced a loose-fitting acrylic tube to help restrict body motion and to reduce MRI artifacts.

### Behavioral responses outside the scanner

All mice learned the association between the visual cue and reward availability inside the mock scanner, and the learning was time (training)-dependent. The average number of rewards earned for the first five conditioning days was 62 ± 21 Cue ON vs 48 ± 8 Cue OFF (*p* = 0.081, Wilcoxon Rank Sum), while for the last five days of conditioning mice responded 212 ± 32 during Cue ON vs 85 ± 10 Cue OFF (*p* < 0.01, Wilcoxon Rank Sum; Supplementary Fig. 1). In contrast, mice injected with a viral vector that lacked channelorhodopsin-2 responded 35 ± 17 during Cue ON vs 30 ± 14 during Cue OFF (*p* = 0.88, Wilcoxon Rank Sum) for the first 5 days of training and 21 ± 14 Cue ON vs 29 ± 14 Cue OFF (*p* = 0.28, Wilcoxon Rank Sum) for the final 5 days.

### Behavioral responses inside the MRI scanner

**Fig. 2:**
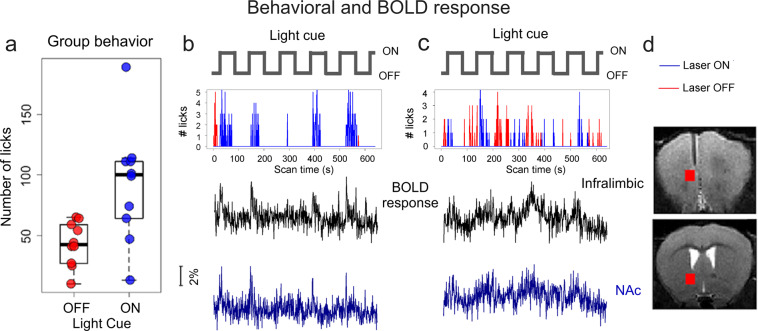
Group behavior (spout licking) and raw time courses of behavioral and corresponding BOLD responses from two mice. **a** Counts of optogenetic stimulation self-administered by 10 mice averaged across the last 2 scan sessions. There was a significant increase in beam breaks during the cue ON versus OFF period (*p* = 0.008, Wilcox Rank Sum test), suggesting that animals had learned the association between the cue and reward availability. **b**, **c** Raster plots of beam breaks from 2 mice during a 640 sec scan session with corresponding BOLD time courses in the infralimbic cortex (the stimulus loci) and the nucleus accumbens (NAc), indicated by the two red blocks in **d**. The blue and red lines in **b** and **c** indicate spout lick during cue ON and OFF respectively. Opto-ICSS was delivered only during cue ON.

All animals learned to self-stimulate inside the real MRI scanner. Supplementary Fig. 2 depicts randomly selected raw raster plots of photo beam breaks from 10 mice in one 10 min run. The temporal occurrence and variability of licking behavior in relation to cue presentation can be readily appreciated. Each mouse was scanned over five days, with 2-3 behavioral runs per day. Data were analyzed and are presented only from the last two scan days after behavioral responding stabilized, where mice licked 92 ± 45 during cue ON vs. 43 ± 17 during cue OFF periods (*p* < 0.01 Wilcoxon Rank Sum) (Fig. 2a).

### BOLD response to self-initiated optogenetic stimulation

Since the delivery of each opto-ICSS pulse was motivated and determined exclusively by the animal, the number of stimuli delivered to each mouse differed. This contrasts with conventional fMRI in which the number and temporal sequence of the stimuli are pre-determined by the experimenter. Figure 2b and c illustrate the temporal behavioral responses from 2 mice, representing a high- and medium- performing animal. Simultaneously acquired BOLD time courses within the opto-ICSS target loci (ILc) and the nucleus accumbens (an ILc projection region) are also illustrated. As can be seen, individual licking events induced appreciable changes in the BOLD signal.

Body motion coinciding with task execution is known to confound the fMRI signal^15^. Since spout-licking was a temporally isolated event, the resulting body motion was brief and primarily time-locked to spout licking; on the other hand, the BOLD response has an intrinsic hemodynamic delay. This temporal characteristic provides the rationale to censor high-motion time points as identified by framewise displacement (FD) calculations^16–18^. As expected, censoring high motion time-points significantly decreased FD (0.038 mm ± 0.01 mm vs. 0.022 mm ± 0.0025 mm; *p* < 0.001 Student’s T-test, paired, one tailed; supplementary Fig. 3), and eliminating correlation between the FD time course and BOLD signal (*r* = 0.21 ± 0.12 vs. *r* = 0.067 ± 0.056; *p* < 0.001 Student’s T-test, paired, one tailed), indicating that the components of the BOLD signal attributed to body motion were properly removed.

### Brain activation to opto-ICSS during Cue ON

**Fig. 3:**
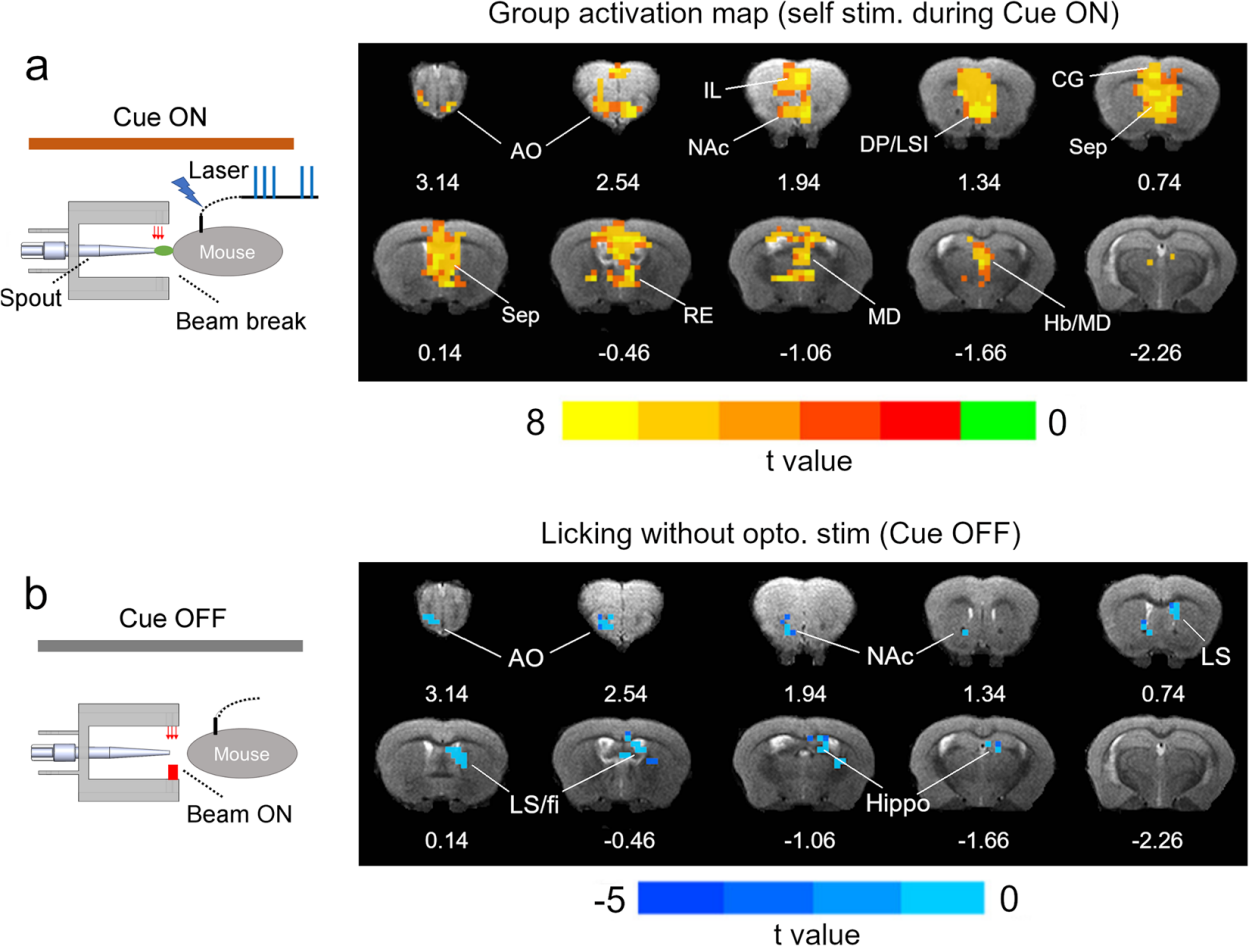
BOLD activation during Cue ON and Cue OFF. **a** Group activation maps following optogenetic self-stimulation during the Cue ON period (*N* = 10, corrected *p* < 0.01). Activated areas include: anterior olfactory nucleus, cingulate cortex (CG1/CG2), prelimbic and infralimbic cortex (IL), nucleus accumbens core (NAc), septum, reuniens thalamic nucleus (RE), medial dorsal (MD) thalamic nucleus and habenula. **b** Group activation maps during Cue OFF period when mice licked the spout in the absence of Opto-ICSS (*N* = 10, corrected *p* < 0.01). Negative BOLD responses were detected in anterior olfactory nucleus (AO), anterior nucleus accumbens (NAc), dorsal and fimbria (fi) hippocampus and lateral septum (LS). Numbers below images are coordinates relative to bregma.

We employed an “event-related” fMRI design approach to derive BOLD activation maps after opto-ICSS^19^: each optogenetic pulse train was treated as an individual “event”, and the resulting “ideal” BOLD response was modeled using a canonical gamma variant function following motion correction. The voxel-wise BOLD response was derived by deconvolving a voxel time course using the modeled BOLD response; the six motion parameters and the cue ON/OFF were treated as the nuance regressors (see Methods). BOLD responses across multiple scan sessions were averaged for each animal, which were then subjected to t-statistics against the null hypothesis to derive activation maps corrected for multiple comparison (Fig. 3a). Opto-ICSS induced activation in a widely distributed network consistent with the underlying anatomical projections of the MPFC^20^, including the cingulate cortex (CG1/CG2), prelimbic (PrL) and infralimbic cortex (IL), piriform cortex, nucleus accumbens (NAc), septal nuclei (sep), dorsal medial striatum (DMS), habenula and midline anterior thalamic nuclei (Hb/MD).

### BOLD activation to spout-licking during Cue OFF

While our study was designed to detect BOLD responses following cue-signaled rewarding brain stimulation, mice also respond, albeit less frequently, during cue OFF periods (Fig. 2a and Supplementary Fig. S2). Even after extensive behavioral training, licking during cue OFF likely led to mild conditioned extinction. Using the same “event-related” data analysis strategy as above, we analyzed BOLD response to licking during the OFF period (without the delivery of opto-ICSS). Negative BOLD signals are seen in the anterior olfactory nucleus, anterior NAc, lateral septum and dorsal hippocampus (Fig. 3b).

## Discussion

We report the results of an awake imaging platform that permitted dynamic fMRI acquisition from mice actively engaged in a goal-directed operant behavior that resulted in optogenetically delivered, rewarding stimulation to the MPFC. Mice learned to respond more for opto-ICSS during cue light ON vs. OFF periods (Fig. 2b) in the otherwise hostile MRI environment, which evoked a robust BOLD response (Figs. 2b and 3a) that was not confounded by behavioral motion (supplementary Fig. 3b). Brain regions activated during opto-ICSS including the cingulate cortex (CG1/CG2), prelimbic and ILc, piriform cortex, nucleus accumbens (NAc), septum, and midline/mediodorsal thalamic nuclei^21^ are consistent with the underlying direct projections of the MPFC. We also detected a negative BOLD response associated with acute extinction (Fig. 3b).

Activation of the NAc is consistent with both human and rodent mesocorticolimbic dopamine circuits and thus reflects the known reinforcing, goal-directed properties of this pathway^21,22^. Activation of the cingulate cortex, which is known to integrate reward and action related information, suggests the engagement of executive control processes in this goal-directed task^23^. The potential role of other identified regions (e.g. piriform cortex, septum) in reward and goal-directed behavior requires further investigation. This pattern of BOLD activation is in contrast to a previous study by Ferenczi et al.^24^, who reported BOLD activation confined within the stimulation loci (ILc). However, a step-function opsin was transfected in that study, which is known to cause prolonged depolarization in contrast to the channelrhodopsin used herein, which has a fast response time and mimics phasic firing of MPFC neurons. Thus, the temporal pattern of MPFC neuron excitation differentially codes activity in its downstream targets.

We detected negative BOLD signals in response to spout licking during cue OFF periods in the absence of optogenetic pulse delivery. Although mice were well trained and learned to associate the contextual light cue and reward availability, we interpret such residual licking during cue OFF periods resulted in acute extinction, and likely accompanied by negative emotions, e.g. disappointment, frustration, negative learning error signal, etc. These observed negative BOLD signals of interest in light of previous research findings. For example, pain (also engendering negative emotion) is known to induce negative BOLD signals in the NAc^25,26^ and lesions of the anterior olfactory nucleus (also identified herein) disrupt extinction learning. Moreover, the negative BOLD signal in the septum and hippocampus is consistent with the notion that the septo-hippocampal system plays an important role in behavioral inhibition and extinction learning^27^. Thus, the present imaging platform is capable of detecting BOLD signal changes associated with cognitive dysregulation and a presumptive negative emotional state in the absence of expected reward delivery, analogous with negative BOLD signals in the ventral tegmental area and striatum in humans in response to events in which a monetary reward is withheld^28^. As for positive rewarding behavior, such negative temporal difference error signal signals cannot be otherwise assessed in the absence of behavioral engagement.

There have been considerable efforts to minimize confounds from anesthesia in rodent fMRI studies^1,24,29–32^. In general, the previously reported methods relied on tight restraint of the animals and extensive habituation training. Moreover, in these studies animals were typically anesthetized before being loaded into the restraint cradles, imaging data were acquired after animals awakened from anesthesia, concerns of residual restraint stress and lingering effects of anesthesia associated with these methodologies remain. A preliminary study reported fMRI of mice performing a task^32^. However, two major technical concerns in imaging awake rodents, namely how to mitigate stress and how to minimize imaging artifacts from motion in a hostile MRI environment, were not addressed. In contrast, to alleviate such concerns, the current study utilized a patented imaging platform issued to NIH^33^, where the key design concept is to alleviate stress associated with awake imaging via positive reinforcement learning and operant training, which intrinsically leads to less motion artifacts.

Another methodological advance incorporated in this platform is the channelrhodopsin viral vector transfection, which allows for the modulation of specific brain circuits and cell types. The development of genetically targeted molecular switches has made it possible to manipulate the activity in defined cell populations and specific projection pathways. Protein channels activated by light or exogenous molecules, can hyperpolarize or depolarize neurons, offering the opportunity to interrogate the causal roles of specific cell populations in behavior. Because of the wide availability of transgenic mice that allows cell-type specific targeting, the mouse is a leading model system for dissecting the neural circuits of the mammalian brain. FMRI offers the opportunity to combine systems-level whole brain readout with modern in-vivo cell biology tools, such as chemogenetic^34^ and optogenetics^35^, to dissect circuit dynamics; and as a noninvasive tool, an fMRI readout from animals is readily translatable into humans.

The whole brain readout combined with cell type- and circuit-specific manipulation tools has the potential to create a paradigm shift in the design of preclinical imaging studies. Critically, since most human fMRI studies are correlational in nature, by employing a common fMRI BOLD signal metric across species, this study provides an important translational platform to unravel causative neural mechanisms of adaptive behaviors in both the healthy naïve animals and in models of neuropsychiatric disorders. For example, the motivation that drives the licking response during cue ON vs OFF, reward anticipation, reward contingency (whether the animal actually received the reward) and how the brain response change as a function of drug use, our study fills an urgently needed technical gap by providing a platform that allows further exploration of the above questions.

Stress associated with head-fixation and the MRI environment is of major concern in awake rodent neuroimaging. Due to a mouse’s small circulating blood volume, it is not practical to longitudinally sample stress hormone levels at various time points during the acclimation protocol and MRI experiments. We instead relied on well-established behavioral signs to gauge stress levels during the behavioral conditioning^36^. A recent imaging study by Tsurugizawa et al.^37^ evaluated stress hormone levels through trunk blood collection, and found that mice did experience a spike of corticosterone during the first day of acclimation, doubling from baseline level, which returned to baseline after 4 days of conditioning. In a pioneer MRI study with awake rats, King et al.^29^ reported that stress hormone levels returned to baseline after 8 days of training. The duration of acclimation reported in this study was substantially longer (18 days compared to 7 and 8 days, respectively). Furthermore, acclimation in the current study was paired with reward delivery (sucrose water and MPFC stimulation) which are expected to further alleviate acute stress in rodents^38^, and was absent in the above two studies. Nevertheless, the behavioral responses inside the MRI scanner were lower than inside the mock scanner, suggesting that the mice might still experience certain levels of stress. Variations in environmental settings may have contributed to this since rodent behaviors are highly sensitive to laboratory settings^39,40^. For example, differences in the tone and pitch of MRI acoustic noises and environmental cues (such as odor) inside and outside the magnet may have contributed to the differential responses. Acclimating mice inside an MRI scanner directly should help minimize such variations. In addition to corticosterone assay, physiological readouts such as respiration rate, heart rate, etc, could be obtained noninvasively or with minimal invasiveness. Such measurement could facilitate quantitative assessment of stress levels, and should be implemented in future studies.

Tissue heating from laser light can induce BOLD signal changes in naïve mice lacking ChR2 expression^31,41^. We performed a control experiments in naïve mice (*n* = 7) to assess potential heating effect. Without expression of ChR2 in the mPFC, these mice could not be conditioned for self-stimulation in response to the visual cue due to the lack of reward association (see Supplementary Fig. S1). These mice received passive light stimulation (block design: 20 s ON, 40 s OFF, see Methods for details). We observed small (<1%), negative BOLD responses in the slice with fiber implantation and in the slice immediately anterior to fiber tip. These negative responses had a slow onset and took 30 s to return to baseline. This response was only marginally significant (*p* < 0.05 prior to statistical correction for multiple comparisons). No significant negative BOLD response was observed at a laser power of 5 mW. For comparison, we also performed identical experiments in awake mice with ChR2 expression. We observed only positive BOLD responses (~4%). These responses had a fast onset and returned to baseline within 10 s after stimulation cessation - typical BOLD response as we would expect. Since neighboring self-administration events last much shorter than 20 s (as shown in Fig. S2), we thus expect that MRI signal changes resulting from tissue heating should be minor in the current study.

Finally, mouse motor cortex of the tongue/jaw region is located in the anteriodorsal part of the brain (approximately + 2.0 mm from bregma)^42^, in close proximity to where the optic fiber was implanted for optogenetic stimulation. Magnetic susceptibility from surgical implants (fiber, dental cement, screws etc. See Supplementary Fig. S6) and procedures caused distortion and signal dropout in EPI images. The impact varied across animals due to the nature of surgery and recovery, and cannot be fully accounted for by the distortion correction algorithm or image post-processing^43^, reducing the statistical power to detect brain activation in this region. Certain activation voxels shown in Fig. 3 overlap with ventricle regions. In addition to remaining motion or physiological artefacts, partial volume effect may have played a role, considering the small size of a mouse brain and the relatively low resolution of EPI images.

In summary, we report an experimental platform that permits awake, whole-brain imaging while a rodent actively engages in a reward task that results in circuitry-specific neuromodulation. While we demonstrated this platform in the context of fMRI, it can also be readily coupled with other imaging technologies such as positron emission tomography, electrophysiology and optical (e.g. fiber photometry) imaging^44^. Finally, although this proof-of-principle study employed a lick operant reinforced by optogenetic brain stimulation, the platform can be readily extended to other paradigms that require active behavioral engagement, e.g. decision-making, reward learning and memory retrieval.

## Methods

### Animal subjects

Adult male (approximately 3 mo.), *C57BL6J* mice (Charles River Labs) weighing 20–25 g were individually housed in a vivarium and maintained on a 12:12 light-dark cycle (lights on at 07:00 AM). Mice had free access to food and water except during training and testing. All procedures were approved by the Animal Care and Use Committee of the Intramural Research Program, National Institute on Drug Abuse (NIDA).

### Viral vectors

The NIDA Optogenetics and Transgenic Technology Core produced adeno-associated virus serotype-1 (AAV) encoding channelorhodopsin-2 (ChR2) and enhanced yellow fluorescent protein (eYFP) mediated by the hSyn promoter from plasmids obtained from the Stanford Optogenetics Innovation Lab; making the vector pAAV1-hSyn-hChR2(H143R)-EYFP at a final viral concentration of roughly 1.8e12 viral genomes/ml. Viral vector pAAV1-EYFP without ChR2 served as a control virus.

### Headpost surgery

Mice were anesthetized using isoflurane and placed into a Kopf stereotaxic apparatus. The coordinates for viral vector injections into the infralimbic cortex were: AP + 1.6 mm, ML 0.2 mm from midline, DV 2.8 mm. 300 nl of the ChR2 expressing viral vector was injected by a microsyringe pump at 50 nl/min, with an additional 10 min delay before removal of the injection needle (34 gauge, beveled). An optical fiber (200 µm core size with numerical aperture of 0.37), constructed as described previously^45^, was implanted 0.2 mm above the injection site and secured to the skull with dental cement (Geristor A and B cement, Denmat #s 4506 and #034522101). Only enough cement was used to secure the fiber ferrule to the skull, avoiding the skull surface in the posterior portion of the skull in the area of the lambda skull fissure which was used to secure a head fixation post. Cylindrical head posts (4.5 mm in diameter and 20 mm in length) were made of methyl-methacrylate and were attached to the skull with dental cement. A layer of acrylic dental cement was added to overcoat the initial cement surface to help support the fiber ferrule and post. After surgery, mice were housed in groups of 2–3 per cage with ad lib food and water.

### Optical stimulation

A laser (473 nm, Opto Engine LLC, Midvale, UT) was connected via a fiber collimator to a 1 meter fiber-connector (Ø200 µm core, 0.34 NA, Opto Engine LLC, Midvale, UT) coupled to a 3 meter long multimodal optical fiber (Ø200 µm core, 0.39 NA, ThorLabs, Newton, NJ), which was attached to the implanted fiber-optic ferrule. The intensity at the end of the patch cable was measured by a power meter (Thorlabs, Newton, NJ) and set at 12 mW. This accounts for a measured 20% power loss due to the coupling of the implanted ferrule, resulting in an estimated 10 mW at the infralimbic cortex. Timing of the laser pulse sequence was controlled by an Arduino Uno R3 microcontrol board (Arduino, Ivrea, Italy) and Processing 3.0 software platform. The TTL laser pulse train (frequency = 25 Hz, number of pulses = 4, pulse duration = 5 ms) was selected based on prior work showing mice learn to self-stimulate under these parameters (Yang and Ikemoto. Rewarding effects of optogenetic stimulation of the medial prefrontal cortex and adjacent regions in mice. SFN abstract, 2014).

### Head-fixation, behavioral monitoring and mock scanner

We manufactured MRI-compatible head-fixation assemblies for behavioral conditioning and MRI experiments as shown in Fig. 1a and supplementary Fig. 4. Materials used for the assemblies were PVC for the body of the assembly (McMaster Car, Cleveland, OH), polyether-ether ketone (PEEK) for the cantilever head-fixing beam and C-clamp (McMaster Car, Cleveland, OH), 1-1/2” OD and 1-1/4” ID clear cast acrylic tube (McMaster Car, Cleveland, OH) mouse body tube, and nylon for the RF surface planar coil holder. Mice were head-fixed onto the assembly by clamping the surgically attached acrylic rod into the cantilever beam and fixing it with nylon thumb screws (Supplementary Fig. S4). To help alleviate stress during conditioning, a customized MRI-compatible treadmill was adopted from optical imaging studies that enabled the mouse to walk or run^46^. The treadmill had no external motor and was driven by the kinetic friction force whenever the mouse chose to walk (Supplementary Fig. S4). The treadmill stopped when the animal stopped walking, with no obvious movement “overshoot”.

The delivery of sucrose or laser light was triggered by a custom-made optical lickometer that sensed the movement of the tongue via a fiber-optic beam break. An LED located outside the magnet emitted a red light (200 µW, 650 nm, Industrial Fiber Optics, Tempe, AZ) aimed about 1 cm from the right side of the mouth, via a fiber optic cable (ø1.0 mm, Industrial Fiber Optics, Tempe, AZ). The light was collimated by a spherical micro lens (ø2.0 mm, 1.1 mm focal length, Newport, Newport, CA) to prevent loss of light intensity due to scattering. Collected light on the left side was collimated into a receive fiber optic cable that transmitted the signal to a coupled phototransistor responsive to 650 nm wavelength light (Supplementary Fig. S4). This optical lickport was activated by interruption of the light path by tongue movement, which was detected by the Arduino microcontroller, and resulted in the delivery of sucrose or optical pulse train. A third fiber optic cable was located in plane with the mouse’s head and orthogonal to the optical lick port. It delivered white light to serve as a visual cue. Behavioral data were collected and analyzed by the Arduino microcontroller, and transmitted to a PC for data storage and postprocessing.

To habituate mice to the acoustic noise and the enclosed space of the MRI scanner, mice were trained in a mock scanner prior to MRI scans^24,32^. The mock scanner was comprised of a 2 foot long, 10 cm wide PVC tube that housed speakers capable of reaching approximately 115 dB similar to that experienced during echo-planar imaging (EPI). EPI sounds were recorded from the scanner and replayed during head-fixed conditioning.

### Behavioral conditioning

Figure 1c summarizes the behavioral conditioning timeline. Prior to surgery, mice were handled for four, 10-minute sessions to habituate to the training environment and experimenter. Animals then underwent viral vector injection and headpost implantation surgery and were allowed to recover for 5 days with pain relief and antibiotic medications delivered.

Animals were subsequently placed on a water restriction schedule for one week, during which they were trained to lick a spout to receive 10% sucrose solution (0.025 ml) in a non-head-fixed position. By the end of the third training day, all mice displayed reward licking behavior. Mice were given one additional hour of free water access after each training session to ensure adequate hydration and were monitored daily for weight, hydration, ruffled fur, and movement based on guidelines established by Guo et al.,2014. If weight dropped below 85% of baseline pre-restriction level, 1 ml of saline was given subcutaneously, and the mouse placed on ad libitum water access until normal weight was restored.

Animals were subsequently trained to respond for 10% sucrose in the head-fixed position. Conditioning sessions lasted for 15 min with a white-light visual cue signaling reward availability introduced. The temporal sequence of conditioning sessions was: (1) 5 min baseline visual cue off period, and (2) 10 min block-design with the visual cue (1 min on: 1 min off) signaling reward availability. An extended OFF period baseline was built into the conditioning paradigm to train the mouse to not expect rewards during the beginning of head fixation. This was implemented for subsequent imaging as it generally takes 15–20 min to load the animal into the apparatus, perform the high-resolution anatomical scans, slice localization and magnetic field shimming prior to functional scan acquisition. To train the mice for the long scanning sessions, the total length of training gradually increased by 15 min every third day for 2 weeks up to a maximum of 60 min, with the baseline length increasing by 5 min, using the same visual cue during block design portions (1 min ON: 1 min OFF).

After the mouse successfully learned the cue ON sucrose reward association in the head-fixed position, water restriction was terminated and infralimbic optogenetic stimulation (Opto-ICSS) was initiated. Laser light stimulation parameters: 25 Hz, 4 pulses, 5 ms pulse duration, nominal power 12 mW (estimated power at the tip of the implanted fiber tip was 10 mW).

Pilot experiments suggested that, even with successful head fixation, movement of the mouse body caused substantial variations in EPI image quality, likely due to magnetic field changes resulting from body movement, a phenomenon demonstrated previously in human fMRI studies^15,16^. During the final stage of training, we thus introduced an acrylic body tube inside the mock scanner to limit movement of the animal’s body, which prevented the mouse from utilizing the treadmill, for 9 days of acclimation prior to fMRI scans. Progressive training sessions lasted 21 days prior to scanning.

### fMRI experimental paradigm

While all animals learned the task outside the magnet, initial introduction to the MRI environment resulted in a decrease in behavioral performance; however, animals re-acclimated to the new environment and performed the task well by the last two of the 5 scan sessions; imaging data are presented only for these last 2 scan sessions. Supplementary Fig. S2 shows the temporal pattern of spout-licking during 5 cycles of 60 s ON and 60 s OFF cue periods from 10 animals during their last scan session. Imaging sessions were separated by at least 3 days.

Of the 15 animals used in this study, five mice were excluded due to poor viral expression and thus poor behavioral response (<30 self-stimulations/scan) resulting in a total of 10 mice that were submitted for group image analyses. FMRI data were analyzed using an event-related design approach (see below). BOLD responses during the cue ON and cue OFF periods were analyzed separately and were subjected to independent statistical comparisons.

Several studies reported MRI signal changes resulting from tissue heating by laser pulses^31,41^. We also performed additional experiments to assess potential heating effects. Seven mice received control virus (pAAV1-EYFP) to the mPFC. These mice could not be conditioned to self-administer optical stimulation, since without ChR2, optical stimulation was not rewarding to these animals. We performed fMRI experiments on these animals by delivering photostimulation in a block-design paradigm: 20 s baseline followed by 5 cycles of 20 s ON 40 s OFF. Laser pulses were applied during the ON period at 1 Hz pulse train. Each pulse train consisted of 4 pulses at 25 Hz, 5 ms pulse duration (identical to self-stimulation). Laser power was 5 and 10 mW. For comparison, we also performed the same experiments on seven mice with ChR2 expression.

### fMRI image acquisition parameters

MRI data were acquired with a Bruker Biospin 9.4 T scanner on a Paravison 6.0.1 platform equipped with an active-shielded gradient coil (Bruker Medizintechnik, Karlsruhe, Germany). A quadrature volume coil (86 mm ID) was used for RF excitation, and a single-turn surface coil (2 cm diameter) was used for MR signal reception. High-resolution T2-weighted anatomical images were acquired using a rapid acquisition with relaxation enhancement (RARE) sequence. Scan parameters are as follows: TR = 2200 ms, effective TE = 35 ms, RARE factor = 8, field of view (FOV) = 30 × 30 mm^2^, matrix size = 256 × 256, with 25 slices acquired at a slice thickness of 0.6 mm. The decussation of the anterior commissure (approximately + 0.14 mm from bregma) appears dark in T2-weighted sagittal anatomical images and was readily identified to serve as a fiducial landmark to standardize slice localization both within and across animals. Functional images were acquired using a single-shot gradient echo echo-planar imaging (EPI) sequence. Scan parameters were as follows: FOV = 25 × 15 mm^2^, matrix size = 96 × 58, TR = 1000 ms, TE = 15 ms, with 15 slices acquired at a slice thickness of 0.6 mm (bregma −4.06 mm to +4.34 mm), covering most of the brain except the olfactory bulb and the cerebellum. The voxels size was 0.26 × 0.26 × 0.6 mm. Data acquisition bandwidth was 250 kHz. Many brain implants are known to cause distortion and signal dropout in GE-EPI images, which is particularly problematic in high field MR scanners. Supplementary Fig. S5 shows representative single-shot GE-EPI images from 2 animals. Signal from ventral structures, including ventral hippocampus, amygdala, ventral tegmental area and part of hypothalamus, is lost due strong susceptibility in these regions. Signal loss from these regions are typical in GE-EPI of the rat and mouse brains. In some animals, we also experienced some signal loss from rostral and caudal dorsal part of the brain due to the implants

Following final MRI data acquisition, animals were perfused transcardially under deep anesthesia (sodium pentobarbital 80 mg/kg, I.P.). Brains were harvested to examine viral expression (see example in Supplementary Fig. S6).

### Statistics and reproducibility

Distortions in EPI images were corrected by using a reversal k-space trajectory method implemented on the Bruker scanner^43^. We applied the “topup” correction algorithm implemented in the FSL software package (V5.1)^47^. fMRI data were analyzed using the ANFI software package (V5.0). Specific steps were as follows: Motion correction and motion parameter estimate was performed using *3dvolreg* function in AFNI. High-resolution anatomical images from one mouse that had the best positioning was chosen as a template. Images from all other mice were then co-registered onto the template using AFNI function *3dAllineate*. Excessive motion in the fMRI time courses were initially removed with *3dDespike*. Euclidean motion was calculated using the six-parameter output motion files from *3dvolreg*. Time points in which Euclidean motion exceeded a 0.075 mm threshold were censored, in addition to the time point before and after to account for temporal variability of motion displacement^15^. The threshold of 0.075 mm was selected because: 1) decreased motion correlation with BOLD activity (supplementary Fig. 3b) 0.212 ± 0.124 Pearson r value to 0.067 ± 0.056 Pearson r value (p = 0.09; one-tailed) for self-stimulation paradigms, and 2) motion larger than 0.075 mm calculated to be associated with a BOLD signal change of 1% or larger along a high-contrast boundary. The threshold of 1% was conservatively chosen to minimize the artifactual effects of motion on the BOLD signal. A cut-off threshold of 80% was applied to motion censoring. Scans in which 20% of time-points were censored were discarded from the study^20^. EPI voxels were resampled to a larger voxel size (0.375 mm × 0.375 mm × 0.6 mm).

BOLD activation was quantified as follows: the actual form of the stimulus (the pattern of light pulses, ie. optogenetic stimulations) was first binned into 1 sec epochs (the same interval of fMRI data acquisition (TR = 1 sec)). The resulting temporal light pulse patterns were convolved with a hemodynamic function (HRF). Previous studies documented fast HRFs in awake rats and mice^48–50^. The HRFs had a short delay (< 1 sec) and short time-to-peak (~ 2 sec), both of which were much shorter than what have been observed in humans. We applied the *waver* function in AFNI package to generate the HRFs with 0 sec delay and 2 sec time-to-peak. Since TR = 1 sec in this study, a delay of less than 1 sec was not feasible in our implementation. Second, the preprocessed fMRI data were detrended using a second order polynomial and deconvolved with the ideal BOLD time course using the six motion parameters (x-, y-, z- translation, yaw-, pitch-, roll- rotation) and the cue ON/OFF pattern as nuisance variables. Time series data points were censored if motion exceeded 0.075 mm as above. Due to the susceptibility of fMRI data to motion artifacts, a conservative approach of motion censoring and motion regression was used to reduce potential Type-I errors. Output data from *3dDeconvolve* was fed into a generalized least squares time series fit using AFNI’s *3dREMLfit* to calculate the correlation, beta-weights and associated t-value in relation to the ideal BOLD response. Beta-weights from the last 2 scan sessions were averaged on a voxel-wise basis. Group activation maps were derived by voxel-wise beta-weights t-test against zero. AFNI function 3dclustsim was used to estimate statistical significance level and to control false discovery rate. An uncorrected *P* < 0.005 with a cluster size of 18 voxels resulted in a *P*_corrected_ < 0.01 was considered significant. Activation maps were overlaid onto high-resolution anatomical images for display.

### Reporting summary

Further information on research design is available in the Nature Research Reporting Summary linked to this article.

## Supplementary information

Supplementary Information

Description of Additional Supplementary Files

Supplementary Data 1

Reporting Summary

## Data Availability

All relevant data are available from the authors upon request. The source data underlying plots shown in Fig. 2 are provided in Supplementary Data 1.
