# Supplementary material for: Whole brain dynamics during optogenetic self-stimulation of the medial prefrontal cortex in mice
Source: Commun Biol. 2021 Jan 14;4:66. doi: 10.1038/s42003-020-01612-x (PMC7809041; doi:10.1038/s42003-020-01612-x)
Supplement: Supplementary file 1 — Supplementary Information [file 42003_2020_1612_MOESM1_ESM.pdf]

Supplementary Figures

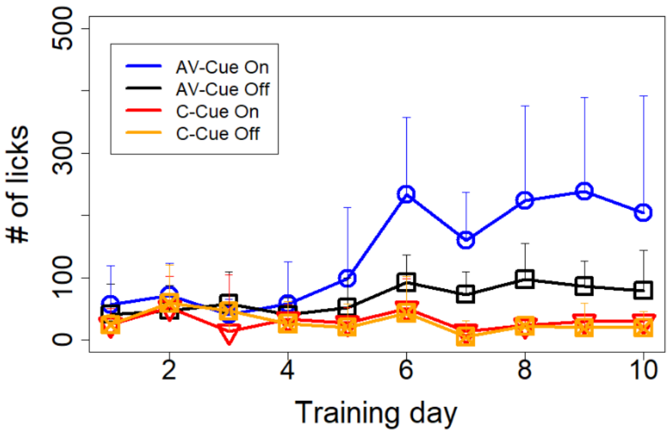

Figure S1.

Figure S1. Operant lick behavior (mean +/-standard deviation) during the first 10 days of training (outside magnet) for optogenetic self-stimulation (opto-ICSS) to the infralimbic cortex. Training took place in the head-fixed position while mice had the opportunity to walk/rest on a self-propelled non-motorized treadmill. Only during the CUE ON period (1 min, alternating with a CUE OFF period), licking the spout resulted in reward-delivery. In mice injected with channelorhodopsin-2 virus (AV), animals learned the association between the cue and reward availability after 5 days of training. In mice injected with control virus (C), behavioral responding was low and indistinguishable between cue ON and OFF. Give stats here!

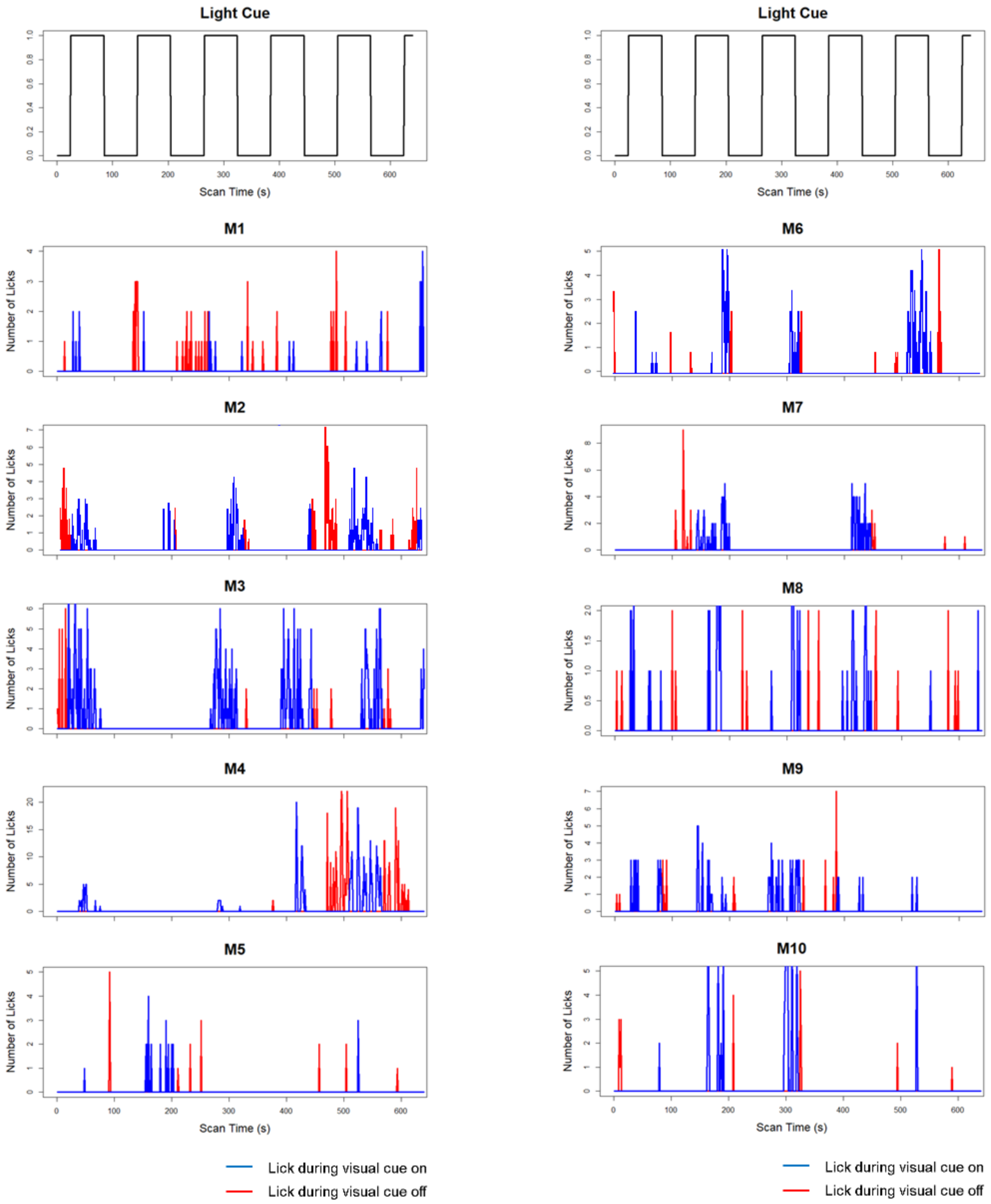

Figure S2.

Figure S2. Temporal patterns of spout-licking during cue ON and OFF periods randomly selected from10 mice during one MRI scan session. Spout-lickings during cue ON resulted in the delivery of opto-ICSS; no laser pulse was delivered during cue OFF.

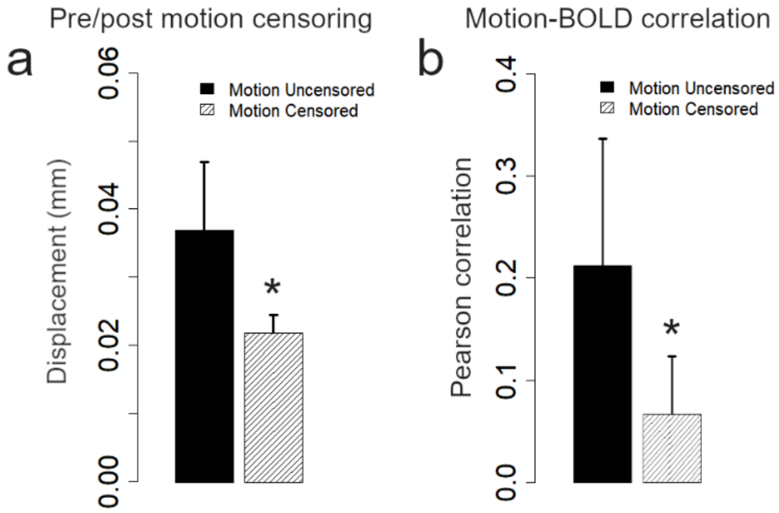

Figure S3

Figure S3. Motion censoring reduced motion effects during Opto-ICSS behavior. (**a)**: Significant reduction in framewise displacement amplitude is seen (*3dvolreg* function in AFNI). (**b**): correlation between motion and BOLD fMRI time courses Motion uncensored: 0.038 mm ± 0.01 mm (mean SD) versus motion censored: 0.022 mm ± 0.0025 mm (p<0.001; Student's T-test, paired, one tailed, n=10). Motion-BOLD correlation ICSS uncensored 0.21 ± 0.12 versus motion-BOLD correlation ICSS censored 0.067 ± 0.056 mm (p<0.001; Student's T-test, paired, one tailed, n=10).

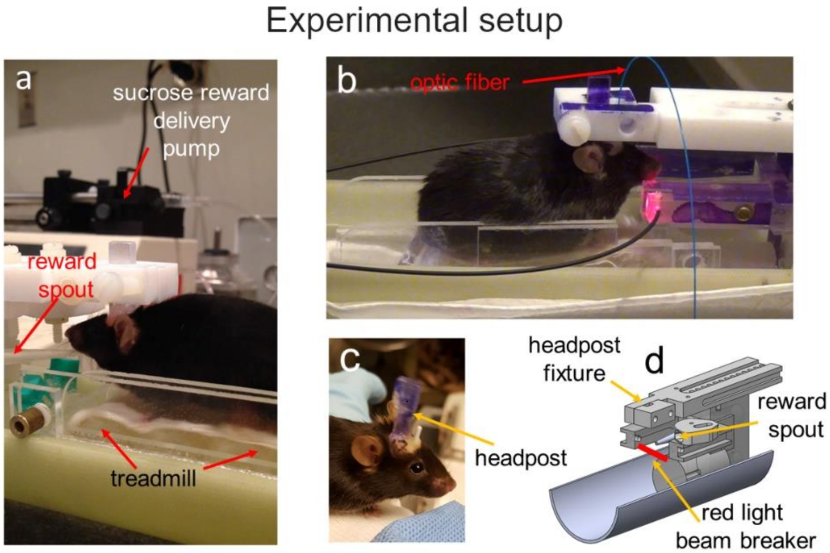

Figure S4.

Figure S4. Experimental setup for behavioral training prior to body tube introduction during fMRI scanning. (**a**) A mouse walking on a non-motorized treadmill with head-fixed post. Sucrose solution (10%) is delivered via a pump each time the animal spout licks. (**b**) The sucrose reward was replaced with optogenetic stimulation. (**c**-**d**) The headpost and the associated fixtures.

Raw EPI images from 2 mice

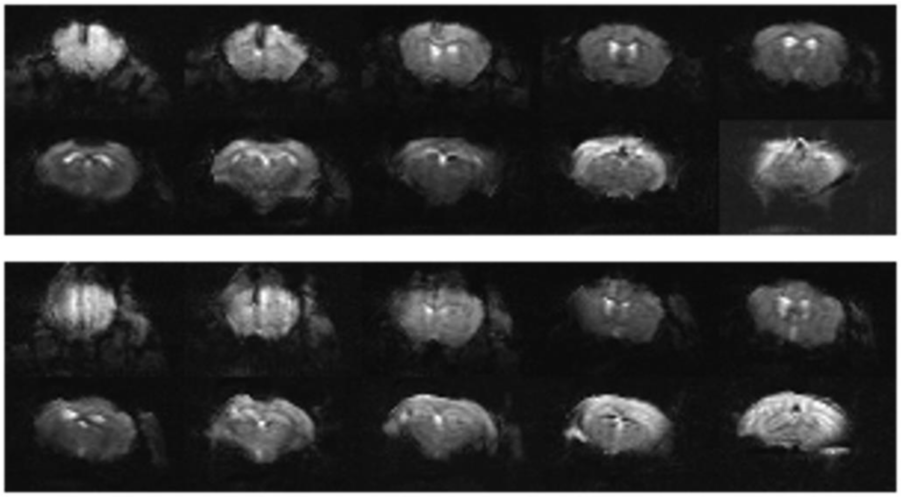

Figure S5.

Figure S5. Single-shot gradient-echo EPI images from 2 representative mice. A headpost fabricated from acrylic was attached to the skull for head-fixation and an optical fiber was implanted aimed above the infralimbic cortex for optogenetic stimulation. MR signal from more ventral structures, including ventral hippocampus, amygdala, ventral tegmental area and part of hypothalamus, is lost due to strong magnetic susceptibility in these regions.

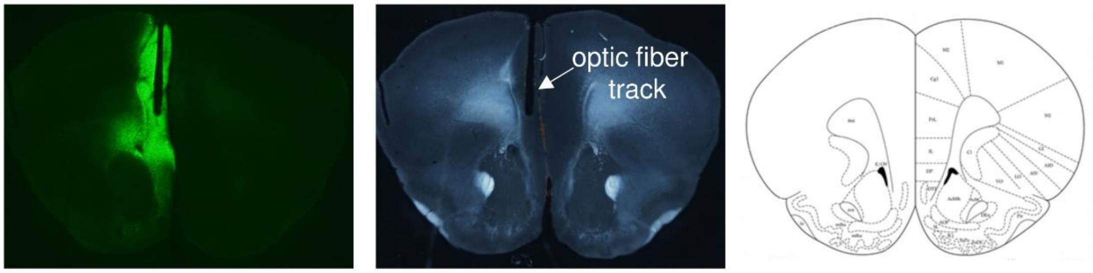

Figure S6.

Figure S6. Example expression of ChR2-eYFP (green) after virus injection into the infralimbic cortex of a representative mouse. The fiber optic tip can be seen just above the infralimbic cortex, located on the corresponding schematic slice.
