# Supplementary material for: Whole brain dynamics during optogenetic self-stimulation of the medial prefrontal cortex in mice
Source: Commun Biol. 2021 Jan 14;4:66. doi: 10.1038/s42003-020-01612-x (PMC7809041; doi:10.1038/s42003-020-01612-x)
Supplement: Supplementary file 2 — Description of Additional Supplementary Files [file 42003_2020_1612_MOESM2_ESM.pdf]

Description of Additional Supplementary Files

File Name: Supplementary Data 1
Description: The source data underlying the graphs and charts in the main figures.
