# Supplementary material for: Whole brain dynamics during optogenetic self-stimulation of the medial prefrontal cortex in mice
Source: Commun Biol. 2021 Jan 14;4:66. doi: 10.1038/s42003-020-01612-x (PMC7809041; doi:10.1038/s42003-020-01612-x)
Supplement: Supplementary file 4 — Reporting Summary [file 42003_2020_1612_MOESM4_ESM.pdf]

nature research

Corresponding author(s): Hanbing Lu

Last updated by author(s): Mar 30, 2020

Reporting Summary

Nature Research wishes to improve the reproducibility of the work that we publish. This form provides structure for consistency and transparency in reporting. For further information on Nature Research policies, see Authors & Referees and the Editorial Policy Checklist.

Statistics

For all statistical analyses, confirm that the following items are present in the figure legend, table legend, main text, or Methods section.

n/a	Confirmed	
☐	☒	The exact sample size (*n*) for each experimental group/condition, given as a discrete number and unit of measurement
☐	☒	A statement on whether measurements were taken from distinct samples or whether the same sample was measured repeatedly
☐	☒	The statistical test(s) used AND whether they are one- or two-sided<br>*Only common tests should be described solely by name; describe more complex techniques in the Methods section.*
☒	☐	A description of all covariates tested
☐	☒	A description of any assumptions or corrections, such as tests of normality and adjustment for multiple comparisons
☐	☒	A full description of the statistical parameters including central tendency (e.g. means) or other basic estimates (e.g. regression coefficient) AND variation (e.g. standard deviation) or associated estimates of uncertainty (e.g. confidence intervals)
☐	☒	For null hypothesis testing, the test statistic (e.g. *F*, *t*, *r*) with confidence intervals, effect sizes, degrees of freedom and *P* value noted<br>*Give P values as exact values whenever suitable.*
☒	☐	For Bayesian analysis, information on the choice of priors and Markov chain Monte Carlo settings
☒	☐	For hierarchical and complex designs, identification of the appropriate level for tests and full reporting of outcomes
☒	☐	Estimates of effect sizes (e.g. Cohen's *d*, Pearson's *r*), indicating how they were calculated

Our web collection on statistics for biologists contains articles on many of the points above.

Software and code

Policy information about availability of computer code

Data collection	FMRI data were acquired using the standard and customized imaging sequences on a Bruker 9.4T MRI scanner
Data analysis	Open source software packages AFNI (Analysis of Functional NeuroImages) and FSL (FMRIB Software Library developed by the Oxford University) were used in the analysis of the data in this study.

For manuscripts utilizing custom algorithms or software that are central to the research but not yet described in published literature, software must be made available to editors/reviewers. We strongly encourage code deposition in a community repository (e.g. GitHub). See the Nature Research guidelines for submitting code & software for further information.

Data

Policy information about availability of data

All manuscripts must include a data availability statement. This statement should provide the following information, where applicable:

- Accession codes, unique identifiers, or web links for publicly available datasets
- A list of figures that have associated raw data
- A description of any restrictions on data availability

The datasets generated during and/or analysed during the current study are available from the corresponding author on reasonable request.

Field-specific reporting

Please select the one below that is the best fit for your research. If you are not sure, read the appropriate sections before making your selection.

☒ Life sciences ☐ Behavioural & social sciences ☐ Ecological, evolutionary & environmental sciences

Life sciences study design

All studies must disclose on these points even when the disclosure is negative.

Sample size	15 mice
Data exclusions	Five mice were excluded due to poor viral expression and poor behavioral response (<30 self-stimulations/scan) resulting in a total of 10 mices.
Replication	Repeated fMRI experiments were performed on the same animals on five different scan days. Two scans/day, resulting in a total of 100 scans.
Randomization	Animals were randomly allocated to the scan sessions.
Blinding	Blinding was not possible, because fMRI tasks relied on self-stimulation, which relied on viral expression (control virus versus channelorhodopsi-2 virus).

Reporting for specific materials, systems and methods

We require information from authors about some types of materials, experimental systems and methods used in many studies. Here, indicate whether each material, system or method listed is relevant to your study. If you are not sure if a list item applies to your research, read the appropriate section before selecting a response.

Materials & experimental systems

n/a	Involved in the study
☐	☐ Antibodies
☐	☐ Eukaryotic cell lines
☐	☐ Palaeontology
☐	☒ Animals and other organisms
☐	☐ Human research participants
☐	☐ Clinical data

Methods

n/a	Involved in the study
☐	☐ ChIP-seq
☐	☐ Flow cytometry
☐	☒ MRI-based neuroimaging

Antibodies

Antibodies used	*Describe all antibodies used in the study; as applicable, provide supplier name, catalog number, clone name, and lot number.*
Validation	*Describe the validation of each primary antibody for the species and application, noting any validation statements on the manufacturer's website, relevant citations, antibody profiles in online databases, or data provided in the manuscript.*

Eukaryotic cell lines

Policy information about cell lines

Cell line source(s)	*State the source of each cell line used.*
Authentication	*Describe the authentication procedures for each cell line used OR declare that none of the cell lines used were authenticated.*
Mycoplasma contamination	*Confirm that all cell lines tested negative for mycoplasma contamination OR describe the results of the testing for mycoplasma contamination OR declare that the cell lines were not tested for mycoplasma contamination.*
Commonly misidentified lines (See ICLAC register)	*Name any commonly misidentified cell lines used in the study and provide a rationale for their use.*

Palaeontology

Specimen provenance	*Provide provenance information for specimens and describe permits that were obtained for the work (including the name of the issuing authority, the date of issue, and any identifying information).*
Specimen deposition	*Indicate where the specimens have been deposited to permit free access by other researchers.*

Dating methods | *If new dates are provided, describe how they were obtained (e.g. collection, storage, sample pretreatment and measurement), where they were obtained (i.e. lab name), the calibration program and the protocol for quality assurance OR state that no new dates are provided.*

☐ Tick this box to confirm that the raw and calibrated dates are available in the paper or in Supplementary Information.

Animals and other organisms

Policy information about studies involving animals; ARRIVE guidelines recommended for reporting animal research

Laboratory animals | Adult C57BL6J mice (20-25g) from Charles River Laboratory delivered to NIDA animal facility.

Wild animals | N/A

Field-collected samples | N/A

Ethics oversight | All procedures were approved by NIDA Animal Care and Use Committee (ACUC), NIH

Note that full information on the approval of the study protocol must also be provided in the manuscript.

Human research participants

Policy information about studies involving human research participants

Population characteristics | *Describe the covariate-relevant population characteristics of the human research participants (e.g. age, gender, genotypic information, past and current diagnosis and treatment categories). If you filled out the behavioural & social sciences study design questions and have nothing to add here, write "See above."*

Recruitment | *Describe how participants were recruited. Outline any potential self-selection bias or other biases that may be present and how these are likely to impact results.*

Ethics oversight | *Identify the organization(s) that approved the study protocol.*

Note that full information on the approval of the study protocol must also be provided in the manuscript.

Clinical data

Policy information about clinical studies

All manuscripts should comply with the ICMJE guidelines for publication of clinical research and a completed CONSORT checklist must be included with all submissions.

Clinical trial registration | *Provide the trial registration number from ClinicalTrials.gov or an equivalent agency.*

Study protocol | *Note where the full trial protocol can be accessed OR if not available, explain why.*

Data collection | *Describe the settings and locales of data collection, noting the time periods of recruitment and data collection.*

Outcomes | *Describe how you pre-defined primary and secondary outcome measures and how you assessed these measures.*

ChIP-seq

Data deposition

☐ Confirm that both raw and final processed data have been deposited in a public database such as GEO.

☐ Confirm that you have deposited or provided access to graph files (e.g. BED files) for the called peaks.

Data access links
May remain private before publication. | *For "Initial submission" or "Revised version" documents, provide reviewer access links. For your "Final submission" document, provide a link to the deposited data.*

Files in database submission | *Provide a list of all files available in the database submission.*

Genome browser session
(e.g. UCSC) | *Provide a link to an anonymized genome browser session for "Initial submission" and "Revised version" documents only, to enable peer review. Write "no longer applicable" for "Final submission" documents.*

Methodology

Replicates | *Describe the experimental replicates, specifying number, type and replicate agreement.*

Sequencing depth | *Describe the sequencing depth for each experiment, providing the total number of reads, uniquely mapped reads, length of reads and whether they were paired- or single-end.*

Antibodies	*Describe the antibodies used for the ChIP-seq experiments; as applicable, provide supplier name, catalog number, clone name, and lot number.*
Peak calling parameters	*Specify the command line program and parameters used for read mapping and peak calling, including the ChIP, control and index files used.*
Data quality	*Describe the methods used to ensure data quality in full detail, including how many peaks are at FDR 5% and above 5-fold enrichment.*
Software	*Describe the software used to collect and analyze the ChIP-seq data. For custom code that has been deposited into a community repository, provide accession details.*

Flow Cytometry

Plots

Confirm that:

☐ The axis labels state the marker and fluorochrome used (e.g. CD4-FITC).

☐ The axis scales are clearly visible. Include numbers along axes only for bottom left plot of group (a 'group' is an analysis of identical markers).

☐ All plots are contour plots with outliers or pseudocolor plots.

☐ A numerical value for number of cells or percentage (with statistics) is provided.

Methodology

Sample preparation	*Describe the sample preparation, detailing the biological source of the cells and any tissue processing steps used.*
Instrument	*Identify the instrument used for data collection, specifying make and model number.*
Software	*Describe the software used to collect and analyze the flow cytometry data. For custom code that has been deposited into a community repository, provide accession details.*
Cell population abundance	*Describe the abundance of the relevant cell populations within post-sort fractions, providing details on the purity of the samples and how it was determined.*
Gating strategy	*Describe the gating strategy used for all relevant experiments, specifying the preliminary FSC/SSC gates of the starting cell population, indicating where boundaries between "positive" and "negative" staining cell populations are defined.*

☐ Tick this box to confirm that a figure exemplifying the gating strategy is provided in the Supplementary Information.

Magnetic resonance imaging

Experimental design

Design type	Event-related design and block design
Design specifications	Self-stimulation paradigm: White-light cue was presented in an ON-OFF block design (5 cycles of 60s ON and 60s OFF). Animals were trained to lick during cue ON period.
Behavioral performance measures	A customized lickometer was constructed to measure mice's lick for reward

Acquisition

Imaging type(s)	Functional and structural MRI
Field strength	9.4 Tesla
Sequence & imaging parameters	Gradient-echo echo planar imaging sequence, TR = 1000 ms, TE = 15 ms, FOV = 25×15 mm, matrix size = 96×58, with 15 slices acquired at a slice thickness of 0.6 mm. The voxels size was 0.26×0.26×0.6 mm. Data acquisition bandwidth was 250 kHz.
Area of acquisition	Whole brain acquisition. The decussation of the anterior commissure (approximately +0.14 mm from bregma) appears dark in T2-weighted sagittal anatomical images, and was readily identified to serve as a fiducial landmark to standardize slice localization both within and across animals.

Diffusion MRI ☐ Used ☒ Not used

Preprocessing

Preprocessing software	AFNI 5.0 and FSL 5.1
Normalization	Linear transformation
Normalization template	High-resolution anatomical images from one mouse had the best positioning was chosen as the template. Images from all other mice were co-registered onto the template.
Noise and artifact removal	Motion in the fMRI time courses were initially removed with 3dDespike in AFNI
Volume censoring	Euclidean motion was calculated using the six-parameter output motion files from 3dvolreg. Time points in which Euclidean motion exceeded a 0.075 mm threshold were censored, in addition to the time point before and after to account for temporal variability of motion displacement. The threshold of 0.075 mm was selected because: 1) decreased motion correlation with BOLD activity (Fig. 4B) 0.212 ± 0.124 Pearson r value to 0.067 ± 0.056 Pearson r value (p=0.09; one-tailed) for self-stimulation paradigms, and 2) motion larger than 0.075 mm calculated to be associated with a BOLD signal change of 1% or larger along a high-contrast boundary. The threshold of 1% was conservatively chosen to minimize the artifactual effects of motion on the BOLD signal.

Statistical modeling & inference

Model type and settings	The behavioral (beam break) data was linearly convolved with an ideal hemodynamic impulse response function using AFNI's waver to derive the ideal BOLD time course. 3dDeconvolve and 3dREMLfit were applied to derive BOLD response with the 6 motion parameters and the cue signal as nuance variables.
Effect(s) tested	Beta-weights from 3dREMLfit were t-tested against 0 to derive the activation map.

Specify type of analysis: ☒ Whole brain ☐ ROI-based ☐ Both

Statistic type for inference (See Eklund et al. 2016)	Cluster-wise. AFNI function 3dclustsim was used to estimate statistical significance level and to control false discovery rate.
Correction	An uncorrected P< 0.005 with a cluster size of 18 voxels resulted in a Pcorrected <0.01 was considered significant.

Models & analysis

n/a	Involved in the study
☐	☐ Functional and/or effective connectivity
☐	☐ Graph analysis
☐	☐ Multivariate modeling or predictive analysis

Functional and/or effective connectivity	*Report the measures of dependence used and the model details (e.g. Pearson correlation, partial correlation, mutual information).*
Graph analysis	*Report the dependent variable and connectivity measure, specifying weighted graph or binarized graph, subject- or group-level, and the global and/or node summaries used (e.g. clustering coefficient, efficiency, etc.).*
Multivariate modeling and predictive analysis	*Specify independent variables, features extraction and dimension reduction, model, training and evaluation metrics.*
